# Deep Brain Stimulation of the H Fields of Forel Alleviates Tics in Tourette Syndrome

**DOI:** 10.3389/fnhum.2017.00308

**Published:** 2017-06-13

**Authors:** Clemens Neudorfer, Faycal El Majdoub, Stefan Hunsche, Klaus Richter, Volker Sturm, Mohammad Maarouf

**Affiliations:** ^1^Department of Stereotaxy and Functional Neurosurgery, Cologne-Merheim Medical Center, Witten/Herdecke UniversityCologne, Germany; ^2^Department of Psychiatry and Psychotherapy, LVR Clinics CologneCologne, Germany; ^3^Department of Neurosurgery, University Hospital of WürzburgWürzburg, Germany

**Keywords:** deep brain stimulation, subthalamus, fields of forel, pallidothalamic fibers, Tourette syndrome, CSTC

## Abstract

The current rationale for target selection in Tourette syndrome revolves around the notion of cortico-basal ganglia circuit involvement in the pathophysiology of the disease. However, despite extensive research, the ideal target for deep brain stimulation (DBS) is still under debate, with many structures being neglected and underexplored. Based on clinical observations and taking into account the prevailing hypotheses of network processing in Tourette syndrome, we chose the fields of Forel, namely field H1, as a target for DBS. The fields of Forel constitute the main link between the striatopallidal system and the thalamocortical network, relaying pallidothalamic projections from core anatomical structures to the thalamic ventral nuclear group. In a retrospective study we investigated two patients suffering from chronic, medically intractable Tourette syndrome who underwent bilateral lead implantation in field H1 of Forel. Clinical scales revealed significant alleviation of tics and comorbid symptoms, namely depression and anxiety, in the postoperative course in both patients.

## Introduction

Tourette syndrome (TS) is the most severe manifestation of a spectrum of related tic disorders that is characterized by multiple, involuntary motor tics and at least one vocal tic persisting for more than 1 year. Pharmacological and psychobehavioral therapy have proven effective treatment options in the management of these cases. In a relatively small percentage of patients that remains refractory to any conventional treatment, surgery may constitute a therapeutic option (Leckman et al., 1998). Starting from the 1960s, ablative procedures, performed in an attempt to control medically intractable, severe tics, sparked the search for the most suitable target in the treatment of TS (Baker, 1962). Neurosurgical interventions varied greatly and included lesioning of the frontal lobe (bimedial frontal leucotomy and prefrontal lobotomy), the limbic system (anterior cingulotomy and limbic leucotomy), the thalamus and subthalamus (Field of Forel and zona incerta) as well as the cerebellum (dentatotomy; Temel and Visser-Vanderwalle, 2004; Figee et al., 2013b). Procedures, however, were often associated with unsatisfactory results and were accompanied by major adverse events such as dystonia and hemiplegia. Vandewalle et al. reported the first application of deep brain stimulation (DBS) in a patient with intractable TS (Vandewalle et al., 1999). The trajectory was based on the thalamotomies carried out by Hassler and Dieckmann (1970, 1973) targeting the centromedian (CM; as component of the intralaminar thalamic nuclei) and ventral oral internal (Voi) thalamic nuclei along with the substantia periventricularis (as component of the midline thalamic nuclei). The medial thalamus remains the most widely studied and used target in TS to date (Visser-Vandewalle et al., 2003; Houeto, 2005; Servello et al., 2008; Porta et al., 2009). Likewise, the globus pallidus internus (GPi) has been credited with considerable importance in tic suppression due to its key role in TS pathophysiology (Welter et al., 2008; Cavanna et al., 2011; Cannon et al., 2012; Zhang et al., 2014). Lesser explored targets include the nucleus accumbens (NA; Kuhn et al., 2007), the anterior limb of the internal capsule (ALIC; Flaherty et al., 2005), the subthalamic nucleus (Martinez-Torres et al., 2009) and the globus pallidus externus (Filho, 2007). Combined treatment approaches using these targets have been performed as well (Shields et al., 2008; Servello et al., 2010). Despite extensive clinical trials, however, uncertainty remains regarding the most suitable target. Recent evaluation of the existing data on DBS for TS showed no significant difference across the main targets, thalamus and GPi (Baldermann et al., 2015). Taking into account the current notion of cortico-basal ganglia (CSTC) circuit involvement in TS pathophysiology and based on our anatomical considerations we successfully performed bilateral DBS of Forel's field H1 in two patients suffering from chronic, treatment refractory TS. Both patients developed TS at a young age and remained refractory to conventional therapy. They exhibited distinct vocal and motor tics and displayed comorbidity of obsessive compulsive disorder (OCD), attention deficit hyperactivity disorder (ADHD) and depression. Following thorough in-patient evaluation and determination of eligibility by a psychiatrist not related to our group, DBS in field H1 of Forel was offered to each patient with the aim to improve the deleterious condition of both patients. Prior to surgery, patients were fully informed about the experimental nature (“individueller Heilversuch”) of this last resort treatment, realistic expectations and all conceivable risks of DBS in this target area. Both patients gave written informed consent in accordance with the Declaration of Helsinki. The IRB of the local ethics committee was informed about the undertaking of a so-called individual treatment attempt (“individueller Heilversuch”) in each case. The decision to perform an individual treatment attempt was made in compliance with German law (Schmitz-Luhn et al., 2012).

## Background

### Rationale and target selection

**Figure 1:**
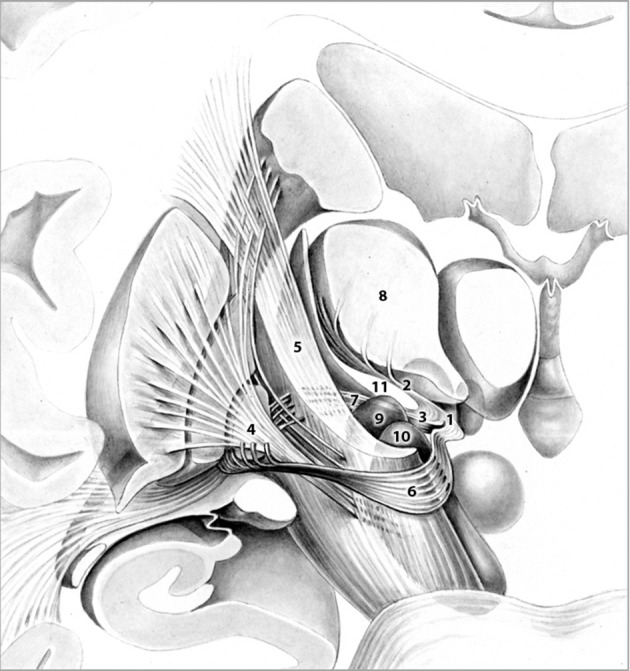
Frontal view of pallidothalamic fibers traversing Forel's fields H (1). H1 (2). and H2 (3). The tract originates from GPi (4). and splits into two major tracts at the level of the internal capsule (5). ansa lenticularis (6). and fasciculus lenticularis (7). Fibers ultimately reunite within Forel's field H and pass through H1 via the fasciculus thalamicus prior to reaching their respective thalamic nuclei (8). Subthalamic nucleus (9). Substantia nigra (10). Zona incerta (11). Adapted with permission from Nieuwenhuys et al. (2008).

Target selection was based on the fact that (1) the fields of Forel (Figure 1) constitute a major convergence of pallidofugal fibers that convey sensorimotor, associative, and limbic information from core anatomical structures to the thalamic ventral nuclear group and occupy a central position within the CSTC system (Alexander and Crutcher, 1990; Gallay et al., 2008; Nieuwenhuys et al., 2008). Fibers originate from the ventromedial aspect of GPi and split into two major tracts—ansa lenticularis (al) and fasciculus lenticularis (fl; field H2 of Forel)—that reunite as field H of Forel in the prerubral field. Pallidal efferents subsequently ascend into the thalamus as Forel's field H1 (Nieuwenhuys et al., 2008). (2) thalamic and pallidal DBS yield significant reduction of tics (Baldermann et al., 2015) and thus, stimulation of pallidothalamic fibers might have an equal if not superior effect on symptoms via orthodromic and antidromic stimulation of both targets. (3) Structures commonly targeted in DBS for TS such as Centromedian-Parafascicular (CM-Pf) Complex, GPi, NA/ALIC, subthalamic nucleus (STN), and ventral anterior and ventrolateral (VA/VL) motor part of the thalamus are located in close vicinity to the H fields and thus, beneficial clinical effects might arise from costimulation of the fiber tracts passing through Forel's fields. (4) Passage of most pallidothalamic fibers through the H fields occurs within a diameter of ~4 mm, H1 thus constitutes an ideal target for DBS (Nauta and Mehler, 1966; Magnin et al., 2006).

The target point was determined based on the Atlas of the Human Brain (Mai et al., 2007). Field H1 of Forel was chosen as the most suitable target, allowing the modification of the surgical trajectory in a way that pallidothalamic fibers were in alignment with the predetermined lead track. It was our intention to place the two distal contacts within H1, whereas proximal contacts targeted the ventral anterior and ventrolateral thalamic nucleus. Thereby we wanted to ensure good clinical benefit both during intraoperative stimulation testing and postoperative stimulation programming. If patients had displayed intolerable side effects during lead implantation the lead could have been retracted from the target point and been placed within the thalamus. Furthermore, if severe adverse events had occurred during follow-up the proximal contacts could have been selected for DBS to ensure clinical benefit while reducing stimulation-related side effects.

### Surgical procedure

Prior to surgery, T1- and T2-contrasted three-dimensional (3D), non-stereotactic magnetic resonance imaging (MRI) scans were obtained in axial and coronal sections with a 1.5 T MRI scanner (Philips Gyroscan Intera, Philips Ltd, Best, The Netherlands). On the day of surgery, a Riechert-Mundinger stereotactic frame (Precisis AG, Walldorf, Germany) was mounted on the patient's head under local anesthesia and stereotactic contrast medium enhanced cranial computed tomography (cCT) scans (SOMATOM Definition Flash, Siemens, Erlangen, Germany) were performed. Registration of both 3D data sets and target planning was subsequently performed using the Praezis Plus Software (Inomed, Emmendingen, Germany). The trajectory was determined as described in the rationale section. To compensate for interindividual anatomical variations, target points were adapted accordingly for each trajectory during stereotactic treatment planning taking into consideration ventricle width, AC-PC distance and hemispheric width. Our target point was located 14–16 mm posterior to the center of the anterior commissure (AC), 1–2 mm ventral to the intercommissural plane and 5–8 mm lateral to the border of the third ventricle.

**Figure 2:**
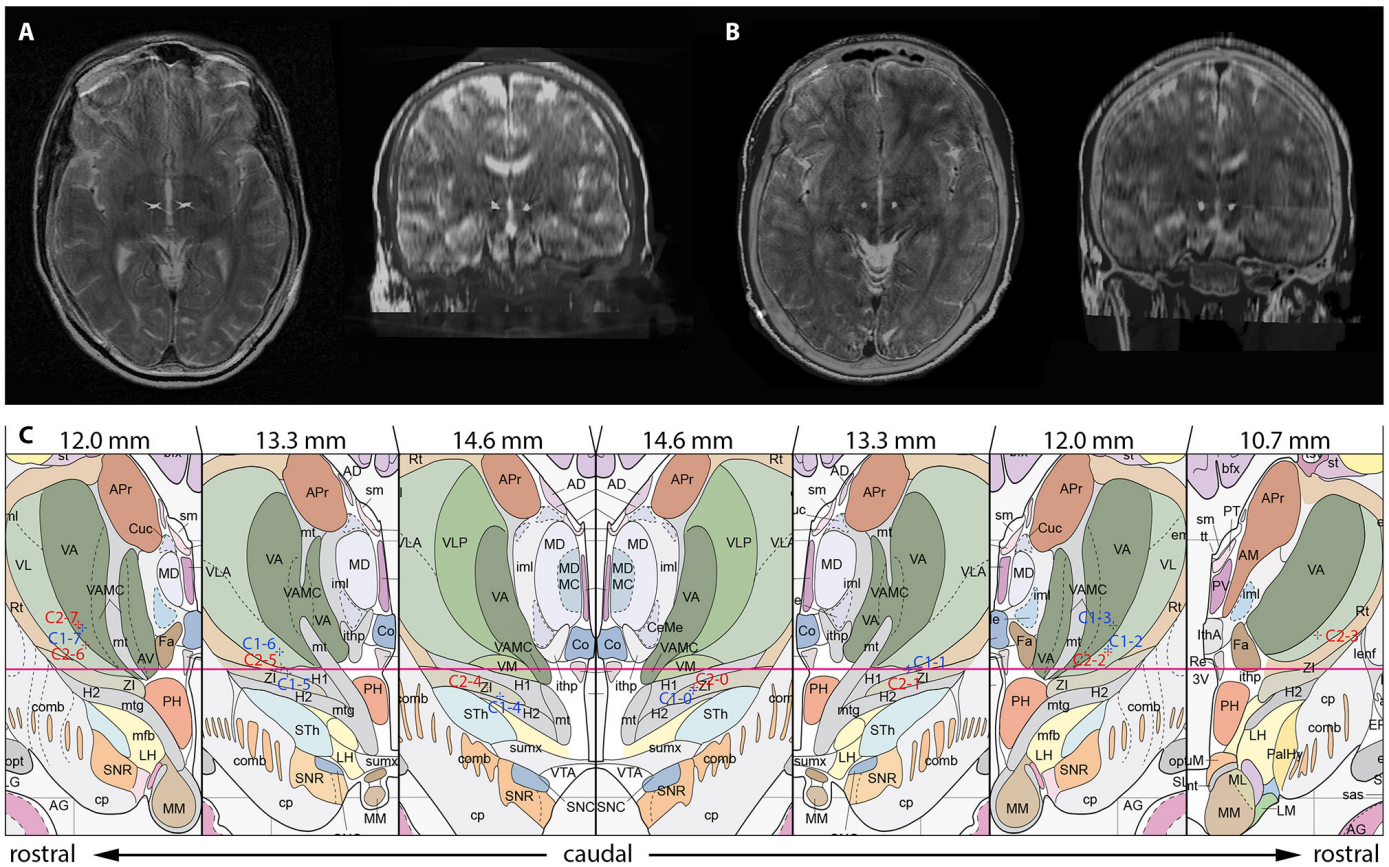
Localization of the most distal contact point within the H fields of Forel on axial and coronal sections in case 1 **(A)** and case 2 **(B)**. **(C)** Anatomical localization of Forel's fields and DBS lead localization according to the Atlas of the Human Brain. Crosshairs represent the centers of active contact points (Case No.–Contact No.) on coronal sections obtained from postoperative 3D stereotactic CCT scans using the O-arm. Effective stereotactic coordinates were conveyed to the Atlas of the Human Brain, considering ventricle width, AC-PC distance and hemispheric width. The coordinates displayed thus represent a transformation of patient coordinates to the standard brain as defined by Mai et al. Actual patient coordinates can be abstracted from Table 1. Adapted with permission from Mai et al. (2007).

After trajectory planning, patients were operated on under local anesthesia. Two quadripolar electrodes (model 3389, Medtronic Inc., Minneapolis, MN, USA) were implanted bilaterally into the predetermined target after burr hole craniostomy and intraoperative stimulation testing was performed to control for acute stimulation induced effects and adverse side effects. Bipolar stimulation between neighboring contacts was performed at fixed frequency of 130 Hz and pulse width of 60 μs; amplitudes were gradually increased from 1.0 to 5.0 V. Intraoperative stimulation testing revealed good clinical benefit in both patients, modification of the electrode track was not necessary. Accurate lead placement was confirmed postoperatively employing 3D stereotactic flat-panel CCT scans using the O-arm (Medtronic Inc., Minneapolis, MN, USA) (Shahlaie et al., 2011). In a subsequent procedure, electrodes were internalized and connected to a programmable implantable pulse generator (IPG; Activa RC, Medtronic Inc., Minneapolis, MN, USA). In the postoperative course, effective stereotactic coordinates were obtained via backward calculation of active contact points from CCT scans using the intercommissural line as a reference (Table 1). Coordinates representing the center of each active contact were then conveyed to the Atlas of the Human Brain, considering again ventricle width, AC-PC distance and hemispheric width (Figure 2C).

**Table 1:** Coordinates of quadripolar leads (model 3389, Medtronic Inc.) targeting the H fields of Forel.

**Patient**	**Contact number**	**Contact localization**	***x***	***y***	***z***	**Contact number**	**Contact localization**	***x***	***y***	***z***
1	0	ZI	−8.8	14.3	−1.8	4	ZI	8.4	14.4	−2.1
	1	H1	−9.3	13.5	0.0	5	H1	8.9	13.5	−0.4
	2	VL	−9.7	12.6	1.7	6	VLA	9.3	12.7	1.4
	3	VA	−10.2	11.8	3.5	7	VA	9.8	11.8	3.3
2	0	ZI	−8.1	14.3	−1.5	4	ZI	8.5	14.4	−2.1
	1	H1	−8.8	13.1	−0.1	5	H1/VLA	8.8	13.5	−0.4
	2	VL	−9.5	11.9	1.4	6	VL	9.3	12.7	1.4
	3	VL	−10.1	10.6	2.7	7	VA	9.8	11.8	3.3

*Coordinates represent the centers of active contacts obtained from postoperative 3D stereotactic CCT scans using the O-arm in both cases. X-coordinates were obtained using the intercommissural line as a reference. Y-coordinates represent the distance from AC to the respective target point. Z-coordinates were obtained using the intercommissural plane as a reference. H1, field H1 of Forel; VA, ventral anterior thalamic nucleus; VL, ventral lateral thalamic nucleus; VLA, ventral lateral anterior thalamic nucleus; ZI, zona incerta*.

### Adjustment of stimulation parameters and pharmacotherapy

Determination of optimal stimulation parameters for each electrode was based on a detailed test-stimulation protocol implemented in the postoperative course. Stimulation parameters initially applied in Forel's field H1 were based on the extensive body of literature describing electrical stimulation of thalamic and pallidal targets (Baldermann et al., 2015). DBS frequency was set at 130 Hz, as is commonly used in hyperkinetic disorders (Montgomery, 2010); the pulse duration was maintained at low values (60 μs), as H1 is a comparatively small target whose volume can be easily encompassed at low stimulation parameters. Monopolar stimulation of each contact was performed successively with gradual increase of the amplitude. Active contacts were eventually selected depending on the best observed clinical benefit on tic reduction and side effects. Stimulation parameters and, if necessary, active contacts were adjusted empirically during follow-up depending on clinical presentation of tics and patient reports (Table 2).

**Table 2:** Stimulation settings following surgery and in the postoperative course.

**Patient No**.	**Time of programming**	**Stimulation settings**
1	Postoperatively	0−, 4−, c+, 60 μs, 130 Hz, 1.0 V
	At discharge	0−, 4−, c+, 60 μs, 130 Hz, 2.0 V
	6 months follow-up	0−, 4−, c+, 60 μs, 130 Hz, 2.1 V
	12 months follow-up	0−, 5−, c+, 60 μs, 130 Hz, 2.9/2.8 V
2	Postoperatively	0−, 4−, c+, 60 μs, 130 Hz, 1.0/1.5 V
	At discharge	0−, 4−, c+, 60 μs, 130 Hz, 2.5 V
	6 months follow-up	0−, 4−, c+, 90 μs, 125 Hz, 1.5 V
		1−, 5−, c+, 60 μs, 125 Hz, 2.9 V
	12 months follow-up	0−, 4−, c+, 60 μs, 125 Hz, 2.2 V
		1−, 5−, c+, 90 μs, 125 Hz, 4.0/3.8 V
	18 months follow-up	0−, 4−, c+, 60 μs, 110 Hz, 2.0 V
		1−, 5−, c+, 90 μs, 110 Hz, 3.5/3.1 V

*Bilateral lead implantation was performed in both patients. Each lead features four contacts with contacts 0 and 4 being located most distal and contacts 3 and 7 most proximal. c, case; pulse duration, [μs]; frequency, [Hz]; voltage [V]*.

Pharmacotherapy (medication and dosage) was maintained in the postoperative course; changes in the pharmacological regimen, were only allowed after 2 months of chronic stimulation. Within the first 6 months of DBS, medication was discontinued in both patients and remained unaltered thereafter.

### Psychiatric and neuropsychological assessment

The primary outcome measure for assessment of the clinical course was reduction of motor and vocal tics as measured by the Yale Global Tic Severity Scale (YGTSS). Yale-Brown Obsessive Compulsive Scale (Y-BOCS), Beck Depression Inventory (BDI) and State Trait Anxiety Inventory (STAI) were used as secondary outcome measures to evaluate comorbidity during follow-up. Modular System of Quality of Life (MSQoL) and Global Assessment of Functioning (GAF) were employed to estimate patients' self-perceived quality of life in the course of DBS (Table 3).

**Table 3:** Baseline characteristics and outcome of H1 stimulation as measured by clinical scales.

**Patient No**.		**YGTSS Vocal**	**YGTSS Motor**	**Impairment**	**YGTSS Total**	**YBOCS Obs**	**YBOCS Com**	**YBOCS Total**	**BDI**	**STAI X1**	**STAI X2**	**MSQoL**	**GAF**
1	Baseline	20	19	40	79	18	14	32	28	72	69	37.08	55
	12 months	2	5	0	7	1	1	2	0	26	25	79.03	91
2	Baseline	14	19	50	83	9	9	18	29	60	72	42.85	39
	12 months	15	17	10	42	9	8	17	14	45	60	63.35	66
	18 months	8	13	10	31	6	9	15	3	37	44	72.98	86

*YGTSS, Yale Global Tic Severity Scale; Y-BOCS, Yale-Brown Obsessive Compulsive Scale; BDI, Beck Depression Inventory; STAI, State Trait Anxiety Inventory; MSQoL, Modular System of Quality of Life; GAF, Global Assessment of Functioning. Persistence of vocal and motor tics at 12 months in case 2 is attributable to complications stemming from non-DBS related medical interventions and work/study related pressure (see case report)*.

### Patients and case histories

#### Case 1

**Figure 3:**
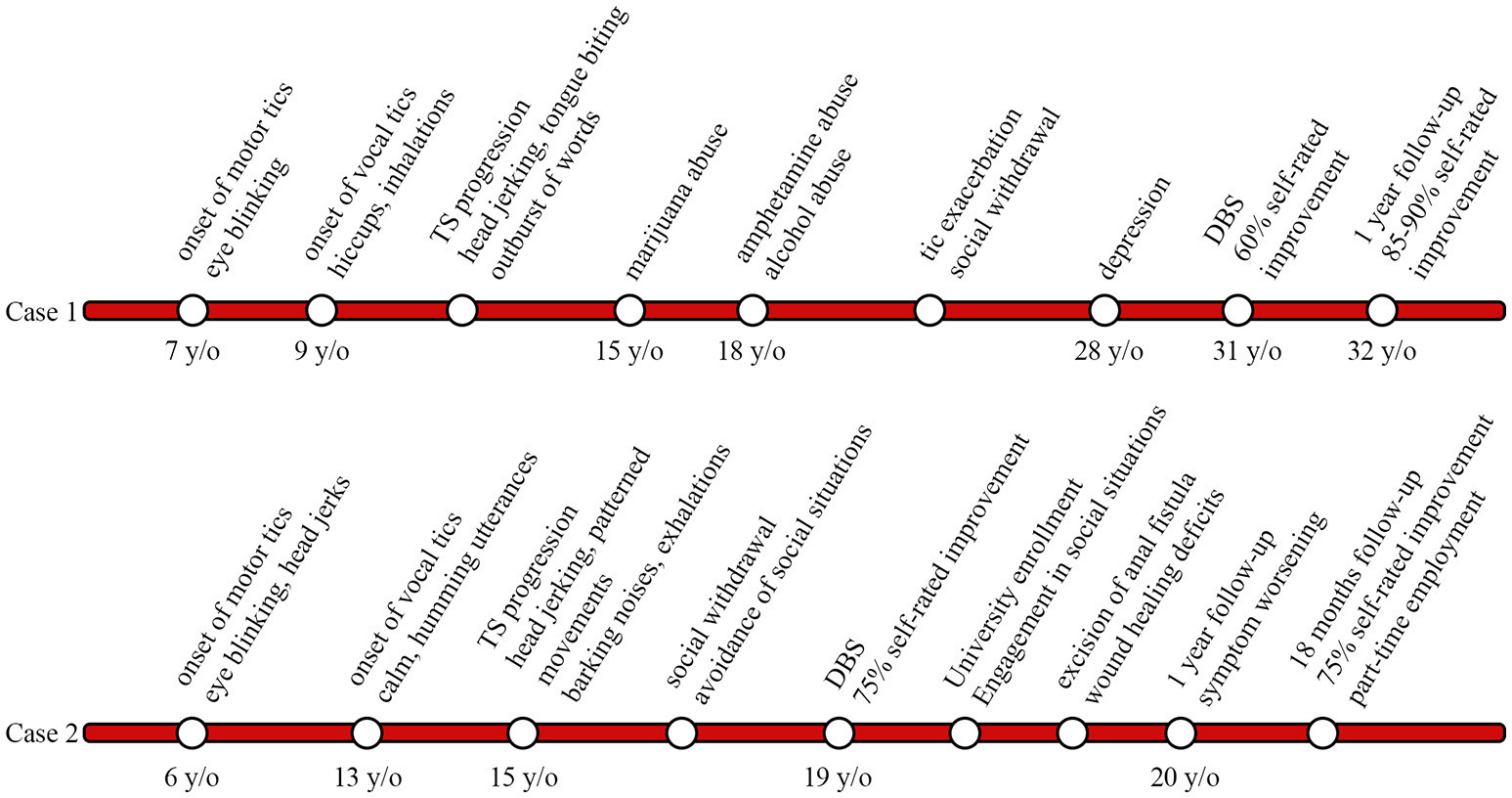
Timeline displaying tic onset, disease progression, deep brain stimulation (DBS) surgery, and follow-up in cases 1 and 2.

A 31-year-old man with a 24-year history of therapy refractory TS and a positive family history of tic disorders presented to our center (Figure 3). Tics initially presented at the age of 7 when he developed simple focal motor tics such as eye blinking and soon thereafter displayed vocal tics including hiccups and exaggerated inhalations by the age of 9; In the course of disease tics became more pronounced in intensity and complexity. Vocal tics expanded to spontaneous outbursts of words; motor tics developed into head jerking movements and self-injurious behavior i.e., tongue biting. Symptom progression led case 1 to take drugs at the age of 15 (marijuana), by the age of 18 he had moved on to amphetamines and alcohol in order to relieve symptoms. Case 1 attended vocational technical school to become a cutting machine operator and soon thereafter was retrained as a system technician. Due to work-related stress and accompanying exacerbation of tics, the patient withdrew socially over the course, avoiding social situations such as grocery shopping. He developed panic attacks and reported episodes of rumination. Three years prior to surgery case 1 fell into a depression. Treatment for tics over the years included, among others, tiapride, diazepam, as well as diverse antipsychotics and antidepressants. Neither drug treatment nor behavioral interventions could alleviate tic severity sufficiently and treatment was eventually discontinued due to intolerable side effects.

Upon admission for H1 DBS the patient's medication included tiapride (200 mg/d). Preoperative psychiatric evaluation disclosed a YGTSS score of 79 with an impairment subscore of 40. Depressive symptoms were found to be stable and he was approved for bilateral H1 stimulation.

Postoperatively, case 1 already noticed marked symptom relieve with the stimulator device being turned off (“insertional effect”) (Figure 2A). In the course of electrical stimulation he experienced good control of tics, which were self-rated at 60% improvement. He noted considerable improvement of complex tics such as tongue biting and was able to discontinue medications. Case 1 was successfully able to resume work and was promoted to shift supervisor within the following year. Depressive and anxiety symptoms subsided entirely, the patient was able to participate in social situations, and furthermore was expecting a child with his girlfriend. At 1-year follow-up he reported nearly complete relief of his tics with 85–90% symptom alleviation. His YGTSS score had improved significantly and was 7 (impairment subscore, 0; Table 3). Stimulation parameters as adjusted during the follow-up period can be obtained from Table 2.

#### Case 2

A 19-year-old young man with a 13-year history of therapy refractory TS, comorbid ADHD and OCD presented to our center for management (Figure 3). Motor tics initially developed at the age of 6 involving simple tics such as eye blinking and mild head jerks. Onset of vocal tics occurred at age 13 and presented as calm, humming utterances. Over the course of the disease, both, motor and vocal tics appeared more frequently with shorter symptom-free intervals and increasing complexity. Vocal tics progressed to complex vocalizations, including high amplitude, barking noises and exaggerated exhalations. Motor tics ultimately manifested as forceful head-jerking tics as well as patterned movements mainly involving the head and trunk. Despite the debilitating symptoms, case 2 attended high school, however due to frequent tic exacerbations he had to transfer schools a total of five times. Symptom progression from age 15 resulted in social withdrawal and avoidance of social situations (e.g., avoidance of crowds or public transportation). Psychiatric evaluation revealed a positive family history of motor tics in the patient's father and maternal uncle. Trials of medication over the years involved tiapride, risperidone, aripiprazole, atomoxetine, tetrazepam, and valproate sodium, none of which led to adequate tic control. Furthermore, cognitive behavioral therapy in out- and in-patient settings hardly yielded any symptom improvement.

On admission the patient's medication included aripiprazole (10 mg/d) and atomoxetine (60 mg/d). Psychiatric evaluation prior to surgery revealed a score of 83 on the YGTSS with an impairment subscore of 50. Comorbid OCD and ADHD were judged to be stable, and thus case 2 was approved for bilateral H1 stimulation.

In the postoperative course stimulation was initiated and continuously optimized (Table 2) (Figure 2B). DBS was well-tolerated and the patient reported marked improvement of tics, which were self-rated at 75% improvement. Furthermore, the patient reported alleviation of obsessive-compulsive symptoms. Within the following months of DBS, case 2 experienced nearly complete relief of symptoms. He was able to discontinue drug therapy and successfully enrolled in university to study economics. Case 2 was able to attend lectures and engage in social situations such as using the public transportation system, activities he deemed impossible prior to surgery. At 1-year follow-up the patient reported worsening of symptoms over the course of stimulation (Table 3). This was attributable to the excision of an anal fistula within months following DBS surgery and wound healing deficits that required resurgery. Furthermore, he reported to be under pressure to perform in his first year of university, which contributed to tic worsening. Despite of worsening of vocal and motor tics over the course of stimulation, overall tic related impairment was judged to be minor. After 18 months of continuous stimulation, the patient's YGTSS score was 31 (impairment subscore, 10). He reported to have taken up part-time employment at a bank.

### Results

Table 3 gives an overview of patients' baseline characteristics and outcome of H1 stimulation during follow-up as measured by clinical scales. Tic severity as assessed by the YGTSS improved significantly upon onset of chronic H1 stimulation and remained greatly reduced during follow-up. Total YGTSS scores improved by 91.1% in case 1; case 2 displayed a reduction of 62.7% at 18 months postoperatively. Tic severity subscores improved by 82.1% (case 1) and 36.4% (case 2), whereas impairment subscores were found to be reduced by 100.0% (case 1) and 83.3% (case 2), respectively at last follow-up. Evaluation of secondary outcome measures revealed significant reduction of depressive symptoms (case 1: 100.0%; case 2: 89.7%). State (STAI-X1) and trait (STAI-X2) anxiety scores dropped in both case 1 (STAI-X1 63.9%; STAI-X2 63.8%) and case 2 (STAI-X1 38.3%; STAI-X2 38.8%) in the postoperative course. Marked improvement of OCD symptom severity was observed in case 1 within the first year of stimulation. Y-BOCS scores dropped by 93.8%, which corresponds to “full response” according to the classification by Pallanti et al. (2002). Only a slight reduction of OCD symptoms, however, could be determined in case 2 (16.7%). Analysis of patients' self-perceived quality of life showed a significant effect of DBS on MSQoL (case 1: 53.1% improvement; case 2: 41.3% improvement) and global functioning. GAF scores changed from baseline 55 (case 1) and 39 (case 2) “serious impairment” to 90 (case 1) and 86 (case 2) “minimal impairment” during follow-up.

## Discussion

While references reporting stereotactic functional interventions in thalamic and pallidal targets for the treatment of TS are extensive, the fields of Forel have only gained little recognition so far. Babel et al. published the only comprehensive report detailing lesioning of the zona incerta and consecutive, partial ablation of Forel's fields H1 and H2 (Babel, 2001). The group's employed target coordinates were located 12 mm posterior to the ventral border of the foramen Monroi (FM), 3–5 mm ventral to the FM-PC line and 9 mm lateral to the border of the third ventricle. Lesioning procedures did not yield any alleviation of tics and/or comorbidities in the postoperative course. Thus, to our knowledge, this is the first study reporting successful tic suppression following functional neurosurgery of the H fields of Forel, in particular field H1, in a retrospective uncontrolled trial of two patients. Successful stimulation can be attributed to the central position of Forel's fields, relaying sensorimotor, associative, and limbic information between core anatomical structures involved in the pathophysiology of TS (Yael et al., 2015). Field H1 can therefore be considered a “bottleneck” both functionally and anatomically (Figure 1). It is embedded within the CSTC circuit at the junction of direct, indirect and hyperdirect pathway, which are believed to be in dysbalance in TS (Alexander et al., 1986; DeLong, 1990; Nambu et al., 2002). Current research suggests that loss of striatal parvalbumin and cholinergic interneurons favors activation of the direct pathway leading to reduced inhibitory control of GPi on downstream thalamic nuclei (Kataoka et al., 2010; Tremblay et al., 2015). Inferring from our observations we speculate that stimulation of field H1 may normalize reduced pallidal output via antidromic stimulation of GPi culminating in the reinstatement of balance within direct, indirect and hyperdirect pathway. Moreover, orthodromic stimulation of downstream thalamic nuclei might restrain overactivity within the thalamocortical network. In two recent studies, DBS of the ventral striatum was shown to reduce functional connectivity within the thalamo-cortico-striatal network in obsessive-compulsive disorder (Figee et al., 2013a; Bahramisharif et al., 2016). A similar mechanism of action may apply to the thalamocortical network following H1 DBS. As the fields of Forel are surrounded by eloquent structures, symptom improvement resulting from co-stimulation of neurons and fibers unrelated to the H fields cannot be excluded.

The thalamus has been studied thoroughly in TS with an extensive body of literature demonstrating suppression of tics, premonitory urges and comorbidities following DBS of CM-Pf, Voi, VA, and VM (Schrock et al., 2015). Given the distinct improvement of symptoms following thalamic stimulation, a key role of the medial thalamus in TS pathophysiology can therefore be assumed. Uniform stimulation comprising all of the aforementioned structures might thus be favorable, however, using conventional DBS methods, coverage of an area that extensive is not feasible. Current targeting strategies aim to circumvent this limitation by placing the electrode at the junction of adjacent thalamic nuclei (Vandewalle et al., 1999; Temel and Visser-Vanderwalle, 2004; Huys et al., 2014; Baldermann et al., 2015). Here, H1 DBS might provide an elegant alternative to this approach allowing stimulation of thalamic afferents within a confined area of ~4 mm in diameter (Nauta and Mehler, 1966; Magnin et al., 2006). As opposed to direct thalamic targeting, DBS of Forel's field H1 can be performed at low stimulation intensities, reducing stimulation related adverse events and maintaining battery economy, respectively (Table 2).

Among functional parallel circuits, the motor pathway is the principal network involved in tic generation (Yael et al., 2015). Premonitory urges as well as comorbid conditions on the other hand are mediated by sensory, associative and limbic pathways. They reside within segregated basal ganglia territories, however, upon leaving GPi, fibers converge within the H fields forming an anatomical “bottleneck” (Parent and Parent, 2004). Alleviation of psychiatric comorbid symptoms might thus be attributable to stimulation of respective functional pathways. Tics and comorbid conditions might also be influenced by stimulation of amygdalothalamic fibers from the ventral amygdalofugal pathway that arise from the rostro-dorso-medial aspect of the amygdala and traverse the substantia innominata. From there, impulses are propagated through Forel's fields and the inferior thalamic peduncle (ithp) to the nucleus fasciculosus and the medial dorsal thalamic nucleus (Aggleton and Mishkin, 1984). Jiménez et al. reported bilateral lead implantation into ithp in patients suffering from therapy refractory OCD and major depressive disorder (MDD; Jiménez et al., 2007, 2013; Jiménez-Ponce et al., 2009). Assessment of OCD symptom severity using the Y-BOCS revealed 51% improvement of obsessions and compulsions after 12 months; by 36 months, scores had dropped by 82.5%. DBS of ithp was performed in one patient suffering from MDD (Jiménez et al., 2005). During chronic stimulation, the Hamilton Depression Rating Scale score decreased from 42 to 6 points; BDI scores decreased from 38 to 11 points. Costimulation of amygdalofugal fibers traversing field H of Forel downstream from ithp might thus contribute to reduction of comorbid symptoms. It should be noted however, that the patient suffering from MDD was explanted at year 4 due to erosion on the head skin without relapse of MDD (Jiménez et al., 2013).

**Figure 4:**
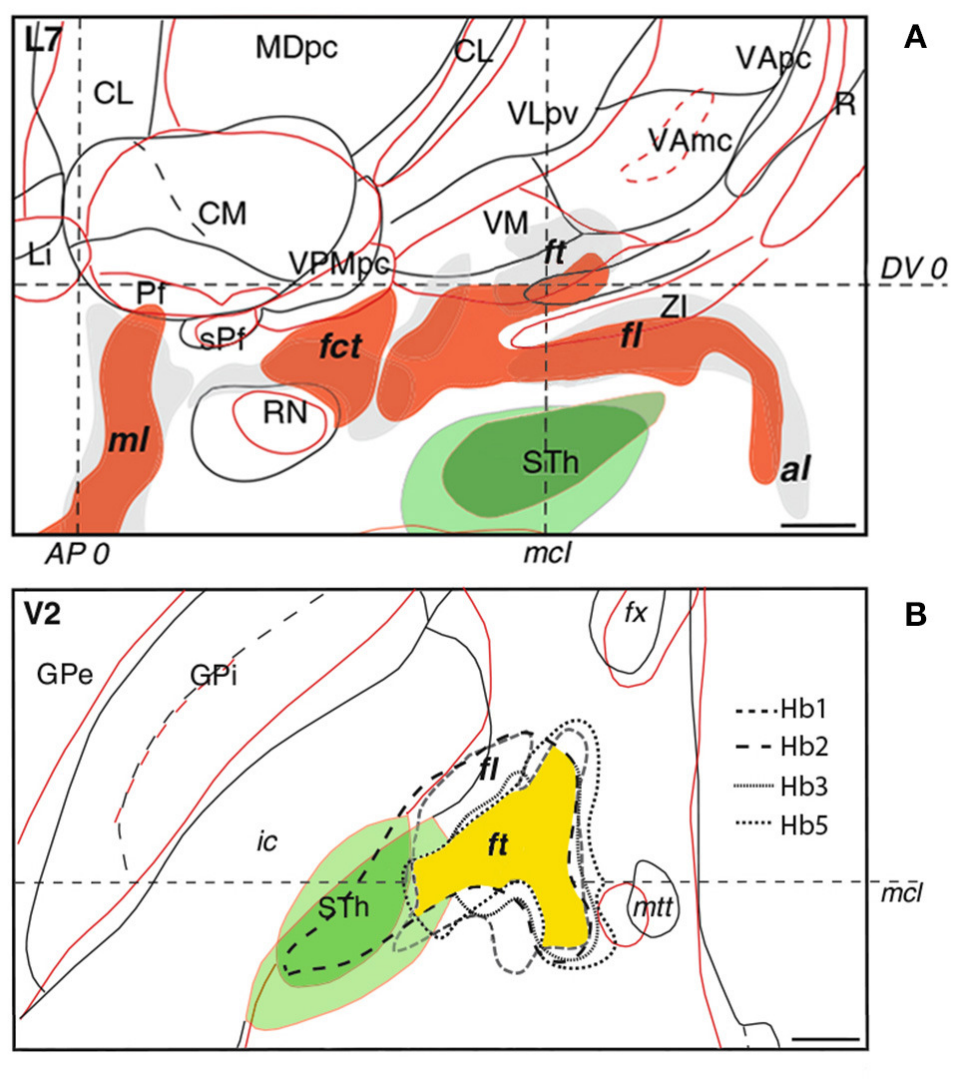
Interindividual variability of the subthalamus as observed in sagittal **(A)** and axial **(B)** histological sections of different brains. In sagittal and axial slices, the *red* and *black/gray* contours and fillings correspond to individual patients. In axial sections, intersubject variability of fasciculus lenticularis (fl) and fasciculus thalamicus (ft) in four patients is represented by differently *dotted lines* with the area of maximal overlap highlighted in *yellow*. The subthalamic nucleus is depicted in *green*. Intercommissural line (DV0). Posterior commissural line (AP0). Midcommissural lines (mcl). Adapted with permission from Gallay et al. (2008).

A drawback in targeting Forel's fields is the lack of anatomical information attainable from MRI and CT scans. Determining optimal lead placement hence proves difficult, especially since the fields of Forel feature considerable interindividual variability. According to Iukharev who studied 100 preparations of coronal diencephalon and midbrain sections, the H fields change from semioval to oval shape in prerubral parts, while pararubral parts display a triangular to rectangular structural arrangement (Iukharev, 1976). Moreover, size and form of Forel's fields differs among patients in relation to anatomical landmarks commonly used during stereotactic procedures, such as posterior commissure (PC), medial and intercommissural plane. As seen on histological preparations of various brains, the fields of Forel overlap to a great extent in anteroposterior and dorsoventral position. Greater differences can be observed in the mediolateral plane with a less marked interindividual mismatch in medial as opposed to lateral portions of Forel's fields (Figure 4; Gallay et al., 2008). These variations are in accordance with overall differences in the mediolateral extent of thalamic and subthalamic areas (Morel, 2007). In order to optimally target H1 and achieve the best clinical benefit during continuous DBS, knowledge of intersubject variability is thus imperative (see also Gallay et al., 2008). The H fields of Forel are surrounded by core anatomical structures including RN, IC, STN, thalamus, cerebellothalamic, and mammillothalamic tract (mtt) that are of considerable importance among functional neural circuits. Costimulation of eloquent structures following incorrect lead placement might consequently limit stimulation intensities and, at worst, rule DBS impossible due to intolerable side effects. In order to avoid stimulation related adverse events and ensure good clinical benefit the target point thus needs to be tailored according to the patient's neuroanatomy. Reference coordinates obtained from stereotactic brain atlases (Mai et al., 2007) allow a rough estimation of Forel's field on MRI scans; consequently, indirect targeting using the surrounding structures (e.g., STN and RN) as landmarks helps to compensate for interindividual neuroanatomical variations. However, given that stereotactic planning occurs on a submillimeter scale, improvements in neuroimaging are required to ensure precise and side effect-free DBS. Possible approaches to improve indirect targeting might involve the visualization of more intricate structures surrounding the H fields. Kerl et al. demonstrated reliable delineation of the zona incerta using 3.0 and 7.0 Tesla (T) MRI systems (Kerl et al., 2012, 2013). Rendering of cerebellothalamic fibers might decrease motor-related adverse events (Kwon et al., 2011). Direct targeting of the fields of Forel and their traversing fiber tracts, as would be desirable, might be achieved using tractography guided approaches. Current major limitations revolving around the application of diffusion tensor imaging (DTI) technology (voxel size, “crossing fiber problem” and seed point dependency among others) however, need to be overcome in order to enable reliable fiber tracking within the H fields of Forel. Reliance on electrophysiological markers as obtained from microelectrode recordings might have been a valuable adjunct to this study as it might have provided comprehensible functional feedback and circumvented the drawbacks concerned with indirect targeting.

## Concluding remarks

In summary, bilateral DBS of Forel's field H1 has been shown to effectively and safely reduce tics and comorbid symptoms in two patients suffering from treatment refractory TS. However, further research and studies including larger study populations are necessary to make a reliable and clear statement about short- and long-term stimulation efficacy in using this target. We advise a cautious approach targeting the fields of Forel, given their marked interindividual variability and eloquent localization among key anatomical structures. Advancements in neuroimaging might provide more reliable tools in the future, allowing better identification and more precise targeting of the H fields, while reducing DBS related adverse events. Implementation of segmented leads might be another valuable supplement as eloquent anatomical structures other than the field's of Forel might be avoided more easily through directed stimulation and thus, stimulation related side effects might be reduced to a minimum.

## Ethics statement

All patients gave written informed consent in accordance with the Declaration of Helsinki prior to surgery. The local ethics committee was informed about the expanded access-trial. In accordance with German law, no separate ethics application and statement by the ethical committee were required for this retrospective study. This, in particular, means that the results of the study have been obtained in a completely anonymous manner. The authors CN, FE, KR, and MM as well as the referring physicians had contact to patients and access to patient's data during medical treatment and follow-up evaluations.

## Author contributions

CN: Designed the study and analyzed the data, wrote the MS, Reviewed and approved the final version. FE, SH, and VS: Analyzed the data, Reviewed and approved the final version. KR: Reviewed and approved the final version. MM: Designed the study and analyzed the data, Reviewed and approved the final version and agreed to be accountable for the work.

### Conflict of interest statement

The authors declare that the research was conducted in the absence of any commercial or financial relationships that could be construed as a potential conflict of interest.
